# Phosphorylation of zinc channel ZIP7 drives MAPK, PI3K and mTOR growth and proliferation signalling†
†Electronic supplementary information (ESI) available: Supplementary figures. See DOI: 10.1039/c6mt00286b



**DOI:** 10.1039/c6mt00286b

**Published:** 2017-02-08

**Authors:** T. Nimmanon, S. Ziliotto, S. Morris, L. Flanagan, K. M. Taylor

**Affiliations:** a Breast Cancer Molecular Pharmacology Group , School of Pharmacy and Pharmaceutical Sciences , Redwood Building , Cardiff University , King Edward VII Avenue , Cardiff , CF10 3NB , UK . Email: taylorkm@cardiff.ac.uk; b Department of Pathology , Phramongkutklao College of Medicine , 315 Ratchawithi Road, Thung Phayathai, Ratchathewi , Bangkok 10400 , Thailand

## Abstract

Zinc is an essential trace element participating in diverse biological processes. Cellular zinc levels are strictly controlled by two families of transport proteins: ZIP channels (SLC39A) and ZnT transporters (SLC30A). ZIP channels increase cytosolic zinc levels by importing zinc into cells or releasing zinc from intracellular stores such as the ER. Among all the 14 human members of the ZIP family, ZIP7 is a gatekeeper of zinc release from intracellular stores, requiring post-translational activation by phosphorylation on residues S275 and S276, resulting in activation of multiple downstream pathways. Employing site-directed mutagenesis, we investigated the importance of these individual serine residues as well as other predicted phosphorylation sites on ZIP7, showing that all four sites are required for maximal ZIP7 activation. Using phosphor-protein arrays, we also discovered the major signalling pathways that were activated as a direct result of ZIP7-mediated zinc release from intracellular stores. These data reveal the role of ZIP7-mediated zinc release from intracellular stores in driving major pathways, such as MAPK, mTOR and PI3K-AKT, involved in providing cell survival and proliferation and often over activated in cancer.

## Abstract 2

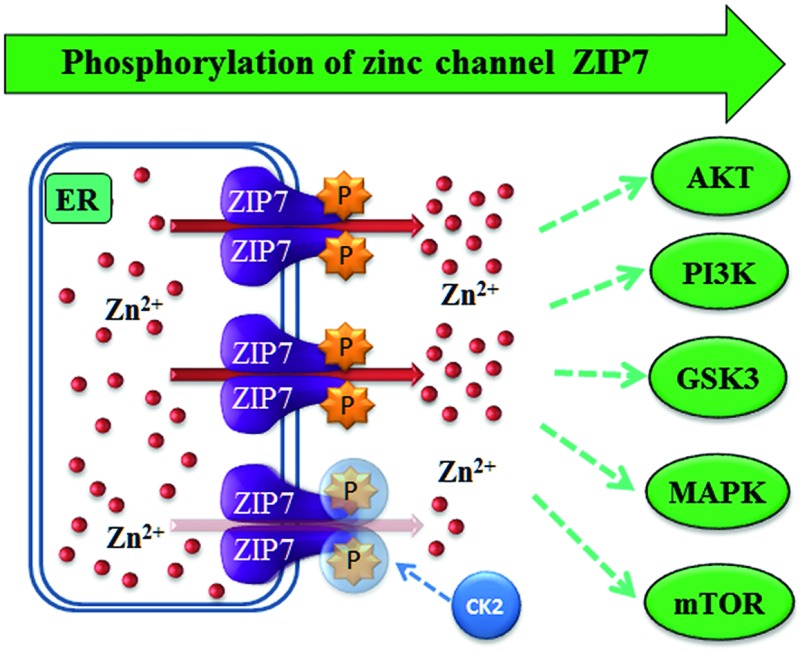
Many important carcinogenesis-related signalling pathways are activated downstream of zinc channel ZIP7-mediated zinc store release.

##

Significance to metallomics

We have previously discovered that zinc transporter ZIP7-mediated zinc release from stores is central to zinc acting as a second messenger and activating numerous signalling pathways known to be important in health and disease. Having explored further the role of ZIP7 phosphorylation and the particular signalling pathways activated by ZIP7-mediated zinc release, we reveal activation of key pathways involved in driving cell survival and proliferation. This discovery together with our new phospho-ZIP7 antibody has increased our understanding of the role of intracellular zinc in maintaining cell growth and provided a new tool to examine active zinc release in biological systems.

## A Introduction

Zinc is the second most abundant trace element in the human body after iron. Zinc is involved in a vast variety of biological processes, being essentially required for the immune system,^1^ the anti-oxidant mechanism,^2^ and neurotransmission.^3^ Importantly, zinc has been shown to act as a second messenger in different cell types, including mast cells^4^ and breast cancer cells.^5^ As such, when a cell is activated by an extracellular stimulus, zinc is released from intracellular stores, such as the ER, resulting in activation of different tyrosine kinase pathways through the inhibitory action of zinc on protein tyrosine phosphatases.^6^

The intracellular level of zinc is tightly controlled by two families of zinc transport proteins: ZIP channels (Zrt- and Irt-like proteins, zinc importers, or SLC39A) and ZnT transporters (zinc exporter or SLC30A).^7^ ZIP channels increase the cytosolic zinc level by mobilising zinc from the extracellular space or intracellular stores, whereas ZnT transporters facilitate zinc transport in the opposite direction.^8^ ZIP channels are divided into 4 subfamilies: gufA (ZIP11), subfamily I (ZIP9), subfamily II (ZIP1–3), and the LIV-1 subfamily (ZIP4–8, 10, and 12–14).^9^ In contrast to other LIV-1 subfamily members, ZIP7 is located on the endoplasmic reticulum membrane and is post-translationally regulated by CK2-mediated phosphorylation on residues S275 and S276.^5^ This ZIP7 phosphorylation results in zinc release from intracellular stores,^5^ which activates multiple tyrosine kinases as well as ERK1/2 and AKT.^10^ Importantly, some of these kinases such as EGFR,^11^ IGF-1R,^12^ and Src^13^ promote the aggressive behaviour of breast cancer cells that have acquired tamoxifen resistance.^14^ Furthermore, the increased activation of these kinases in these tamoxifen-resistant cells has been attributed to increased activation of ZIP7 and the subsequent higher level of intracellular zinc,^10^ linking ZIP7 to the mechanism of acquiring tamoxifen resistance in breast cancer.^15^ Additionally, given the ubiquitous expression of ZIP7 in the human body,^16^ its location on ER zinc stores,^16,17^ and its activation mechanism by phosphorylation,^5^ ZIP7 has been suitably designated as “a hub for tyrosine kinase activation”.^18^

In light of this information, we developed a monoclonal antibody that recognises ZIP7 specifically when phosphorylated on residues S275 and S276 and are using this to determine the downstream targets of ZIP7-mediated zinc release. Upon cellular exposure to an external stimulus, ZIP7 is activated by phosphorylation within 2 minutes, and AKT is activated within 5 minutes in ZIP7-transfected MCF-7 cells.^5^ In this present study, we characterise this new pZIP7 antibody to confirm it recognises the phosphorylated form of ZIP7 and assess its usefulness to detect ZIP7 activation in cells. Employing this antibody, we next determine the importance of the individual residues S275 and S276, as well as other potential recently discovered phosphorylation sites, S293 and T294, in ZIP7 function, revealing that all four residues are required for maximal activation of ZIP7. Furthermore, we investigate the multiple kinases phosphorylated downstream of ZIP7-mediated zinc release, employing three different types of phospho-protein antibody arrays, confirming activation of three major signalling pathways, MAPK, PI3K-AKT and mTOR, as the major downstream targets of ZIP7. This data reveals new downstream targets of ZIP7-mediated zinc release and explains its role in driving cell proliferation and survival.

## B Materials and methods

### Cell preparation, treatment and transfection

MCF-7 cells were cultured as previously described.^10^ Treatments used were 20 μM zinc plus 10 μM sodium pyrithione (zinc ionophore, Sigma-Aldrich, H3261) and 10 ng ml^–1^ EGF and 500 nM ionomycin (calcium ionophore, Sigma-Aldrich, I3909). The generation of SLC39A7 in a pcDNA3.1/V5-His-TOPO vector has been described.^16^ Site-directed mutagenesis was performed to create ZIP7 mutants (S275D/S276D, S275A, S276A, S293A, and T294A), and confirmed by sequencing (Fig. S1A, ESI†). The ZIP7 S275A/S276A mutant has previously been described.^5^ Cells were transfected with Lipofectamine-2000 (Life Technologies) according to the manufacturer's instruction. Briefly, cells grown on 35 mm dishes with 70–90% confluence were transfected with 3.5 μg of DNA and 27.5 μl of the reagent in the antibiotic-free medium. Robust transfection of the mutants was confirmed using V5 immunofluorescence (Fig. S1B, ESI†).

### Antibodies

Rabbit total ZIP7^10^ and mouse monoclonal pZIP7 (S275/S276, Merck, MABS 1262) antibodies have been developed in house. Other antibodies used were pAKT (S473, rabbit, 9271), pGSK-3b (S9, rabbit, 9336), pCREB (S133, rabbit, 9198), p-p38 MAPK (Y180/Y182, rabbit), p-p70 S6 (T421/S424, rabbit, 9204), and pWNK1 (T60, rabbit, 4946) antibodies from Cell Signaling Technology; p-p53 (S392, rabbit, SAB4503954) and β-actin (A5316) antibodies from Sigma-Aldrich; V5 (rabbit, SC-83849-R), and pSTAT5 (S726, rabbit, SC-12893) from Santa Cruz Biotechnology; V5 (mouse, 46-0705) from Invitrogen; and V5 (rabbit, Ab9116) from Abcam.

### Fluorescent microscopy and FACS analysis

1 × 10^5^ cells were grown on 0.17 mm thick coverslips for 5–7 days prior to transfection. Coverslips were fixed and processed as previously described.^19^ Antibodies used for immunofluorescence were pZIP7 (1/200) and V5 (1/1000). For FACS analysis using BD FACSVerse, cells were loaded with 5 μM Fluozin-3AM (Invitrogen) for 30 min at 37 °C, followed by 30 min recovery in medium. FACS data were analysed using Flowing Software version 2.50 (Turku Centre for Biotechnology).

### Blotting, immunoprecipitation and arrays

Cells were harvested, washed with PBS, lysed for 1 hour at 4 °C with lysis buffer pH7.6 (50 mM Tris, 150 mM NaCl, 5 mM EGTA and 1% Triton X-100) with a protease inhibitor cocktail for mammalian cells (Sigma-Aldrich, P8340) and phosphatase inhibitors (2 mM sodium orthovanadate and 50 mM sodium fluoride). Western blotting results of 20 μg per lane from three separate experiments were normalised to β-actin values. All primary antibodies were used at a 1/1000 dilution. Antibody arrays used were the human phospho-RTK (ARY001B), phospho-kinase (ARY003B), and phospho-MAPK (ARY002B) arrays from R&D Systems. Each membrane was applied with 300 μg cell lysate according to manufacturer's instructions. Signal intensities were determined by densitometric analysis using Alpha DigiDoc version 4.10. Heat maps were generated using a GENE-E matrix visualization and analysis platform (The Broad Institute).

### Statistics

Statistical analysis was performed using either Student's *t*-test or ANOVA with *Post-hoc* Dunnett tests. Significance was assumed with * = *p* < 0.05, * = *p* < 0.01, and *** = *p* < 0.001. Error bars are standard errors from at least three different experiments.

## C Results

### The pZIP7 antibody recognises phosphorylated and activated ZIP7

**Fig. 1:**
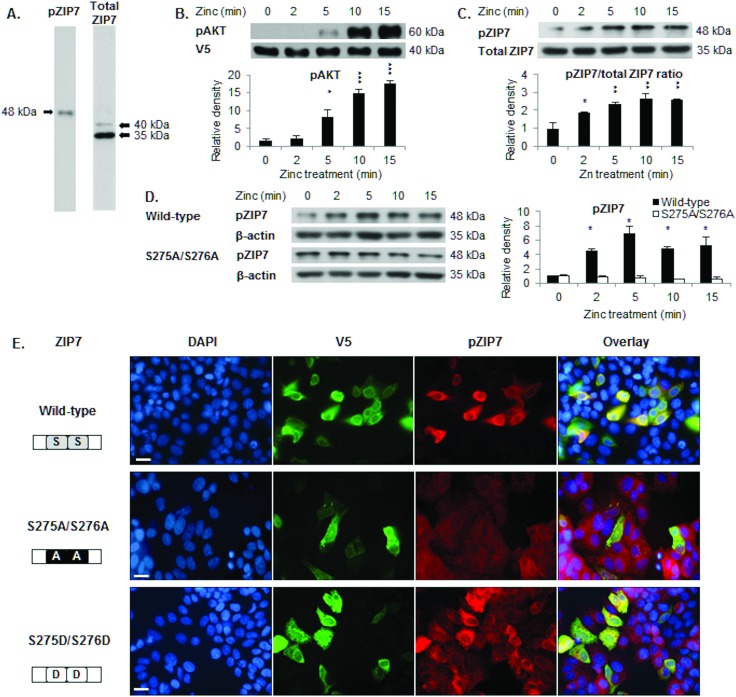
Recognition of ZIP7 activation by the pZIP7 (S275/S276) antibody. (A) Immunoblotting in MCF-7 cells using the pS275/S276 ZIP7 antibody detects a band at 48 kDa, compared to bands of 40 and 35 kDa for the total ZIP7 antibody. (B) Immunoblotting of pS473 AKT in MCF-7 cells transfected with wild-type ZIP7 demonstrates AKT activation after 5 minutes of zinc treatment. (C) Immunoblotting in MCF-7 cells transfected with wild-type ZIP7 shows a pS275 S276 ZIP7 (48 kDa) to total ZIP7 (35 kDa) ratio demonstrating ZIP7 phosphorylation after 2 minutes of zinc treatment. (D) Immunoblotting using the pS275/S276 ZIP7 antibody demonstrates ZIP7 phosphorylation on residues S275 and S276 (48 kDa) after 2 minutes of zinc treatment in MCF-7 cells transfected with wild-type ZIP7, in contrast to cells transfected with the ZIP7 S275A/S276A mutant. (E) Immunostaining using V5 and pS275/S276 ZIP7 antibodies, which were conjugated to alexa fluor 488 (green) and 594 (red), respectively, reveals that the pZIP7 antibody recognises 40% cells transfected with ZIP7 wild-type, 100% cells transfected with ZIP7 S275D/S276D, and 0% cells transfected with ZIP7 S275A/S276A mutants. Statistical significance is compared to time 0. * = *p* < 0.05, ** = *p* < 0.01, and *** = *p* < 0.001. Scale bar, 12 μm.

We have developed a pZIP7 antibody (Fig. S1C, ESI†) to recognise ZIP7 specifically when phosphorylated on residues S275 and S276, a modification of ZIP7 that triggers the zinc transport function of ZIP7.^5^ Western blotting was performed and results obtained with pZIP7 and total ZIP7 antibodies were compared. The total ZIP7 antibody recognised protein bands at 40 kDa and 35 kDa (Fig. 1A). In contrast to the total ZIP7 antibody, the pZIP7 antibody recognised a band at 48 kDa (Fig. 1A), consistent with a mobility shift due to phosphorylation. We examined the protein sequence of ZIP7 for any additional potential phosphorylation sites and found four potential sites according to mass spectrometry-based proteomic studies: S275, S276, S293, and T294 (Table 1).

**Table 1:** Predicted phosphorylation sites on the loop between TM3 and TM4 of ZIP7. Discovery of potential phosphorylation sites in ZIP7 using PhosphoNET,^20^ PHOSIDA (Max Planck Institute of Biochemistry)^21^ and PhosphoSitePlus.^22^ The underlined residue corresponds to the predicted site. MS, mass spectrometry

Site	Sequence	Number of MS studies	Predicted kinases
S275^5^	RSTKEKQSSEEEEKE	13	CK2
S276^5^	STKEKQSSEEEEKET	14	CK2
S293	VQKRRGGSTVPKDGP	1^23^	MAPKAPK2–3
T294	QKRRGGSTVPKDGPV	1^23^	PIM1–3

We next confirmed previous observations that zinc treatment of ZIP7-transfected cells increases ZIP7-mediated zinc release within 5 minutes as judged by AKT activation^5^ (Fig. 1B) and that the pZIP7/ZIP7 ratio (Fig. 1C) or the pZIP7 levels normalised to β-actin (Fig. 1D) increased from 2 minutes, consistent with ZIP7 serine phosphorylation prior to activation of AKT.^5^ In contrast, cells transfected with the ZIP7 S275A/S276A mutant showed no activation of pZIP7 after zinc treatment (Fig. 1D).

To determine the specificity of this antibody for activated ZIP7, immunofluorescence was performed on cells transfected with either ZIP7 wild-type or ZIP7 S275A/S276A (phosphoablative) and S275D/S276D (phosphomimetic) mutants, which contain a C-terminal V5 tag. Forty percent of cells transfected with wild-type ZIP7 stained positive for pZIP7 (Fig. 1E) compared to 0% when transfected with the ZIP7 S275A/S276A mutant, demonstrating that the antibody only recognised these residues when they were phosphorylated. In contrast, almost 100% of cells transfected with the ZIP7 S275D/S276D mutant stained positive for pZIP7 (Fig. 1E). These findings confirm that the pZIP7 antibody recognises ZIP7 only when residues S275 and S276 are phosphorylated.

### S275, S276, S293, and T294 are required for ZIP7 maximal activation

**Fig. 2:**
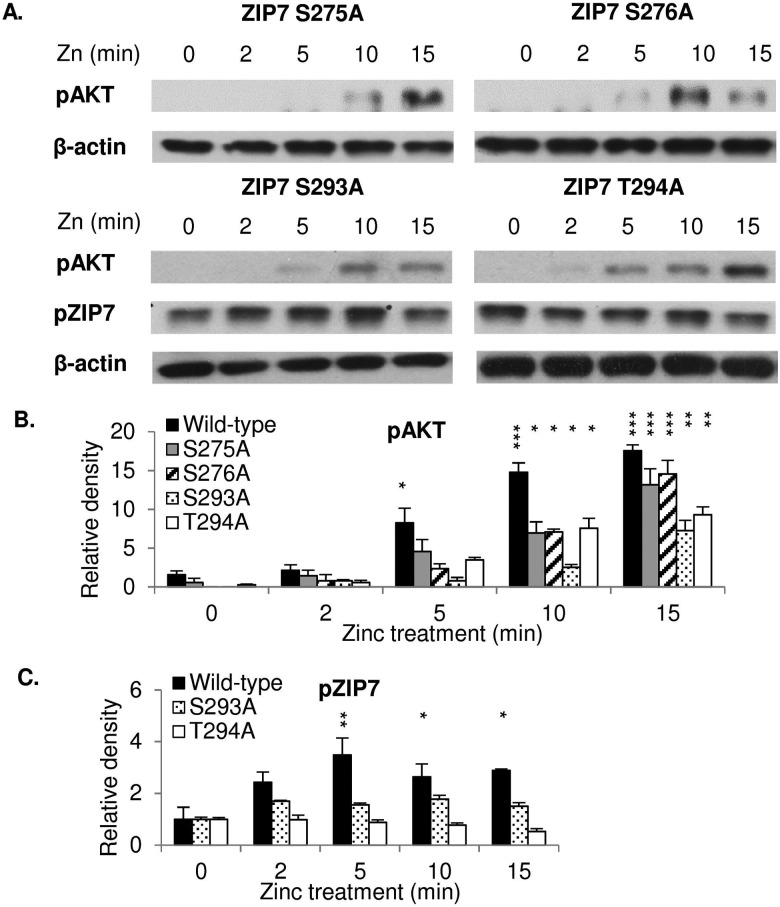
Requirement of S275, S276, S293, and T294 for maximal ZIP7 activation. Immunoblotting using pS473 AKT and pS275/S276 ZIP7 antibodies was performed in MCF-7 cells transfected with ZIP7 S275A, S276A, S293A, and T294A mutants and treated with zinc. Representative blots (A) and densitometric data for pS473 AKT (B) and pS275/S276 zip7 (C) are presented. Statistical significance is compared to time 0 of each construct. * = *p* < 0.05, ** = *p* < 0.01, and *** = *p* < 0.001.

To investigate whether the individual residues S275, S276, S293, and T294 were required for ZIP7 activation, cells were transfected with appropriate mutants and tested for their ability to initiate a ZIP7-mediated zinc release. Cells transfected with these ZIP7 mutants required 10 minutes after zinc treatment in order to activate AKT (Fig. 2A and B), in contrast to cells transfected with wild-type ZIP7, which were able to activate AKT within 5 minutes after zinc treatment^5^ (Fig. 1B), suggesting that all these residues were required for the maximal activation of ZIP7. Furthermore, the ZIP7 S293A and T294A mutants did not appear to activate ZIP7 during the 15 minutes of zinc treatment (Fig. 2A and C), suggesting that residues S293 and T294 may play a role in ZIP7 activation. However, both these mutants had increased ZIP7 phosphorylation before any zinc treatment (Fig. 2A) when compared to the wild type ZIP7 (Fig. 1C and D), which may be suggestive of an inhibitory role of S293 and T294 in the initiation of ZIP7 phosphorylation on residues S275 and S276. This needs further examination.

### Investigating downstream targets of ZIP7-mediated zinc release

**Fig. 3:**
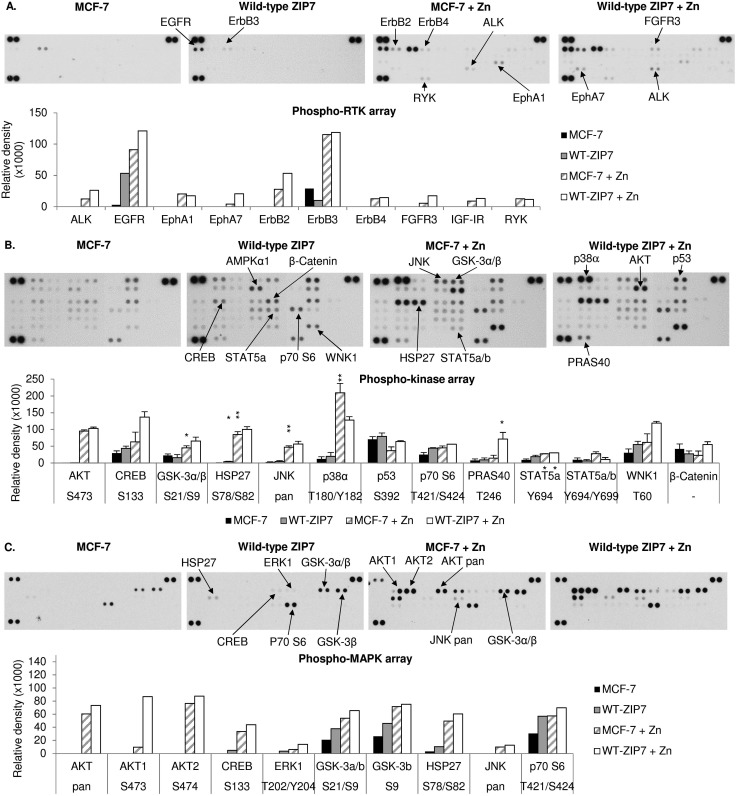
Activation of cellular kinases by ZIP7 overexpression or zinc treatment. MCF-7 cells were transfected with or without wild-type ZIP7 and treated with or without zinc for 10 minutes. Tyrosine phosphorylation of selected RTKs and site-specific phosphorylation of selected kinases were determined using the phospho-RTK (A), phospho-kinase (B), and phospho-MAPK (C) antibody arrays (R&D systems). Signals for each kinase are presented as a pair of duplicate spots, with three pairs of dark reference spots on the upper left, upper right, and lower left corners for alignment. The kinases that show any conspicuous changes in phosphorylation (>10 000 density units) in the non-transfected zinc-treated cells (MCF-7 + Zn) or the transfected non-treated cells (wt-ZIP7) when compared to the non-transfected and non-treated cells (MCF-7) are indicated. In addition, the kinases that show any conspicuous changes in phosphorylation in the transfected zinc-treated cells (wt-ZIP7 + Zn) when compared to the non-transfected zinc-treated cells (MCF-7 + Zn) are also indicated. Average densitometric values for these kinases are shown in bar graphs. The experiments were performed once for the phospho-RTK and phospho-MAPK arrays and twice for the phospho-kinase arrays. Statistical significance compares wt-ZIP7 or MCF-7 + Zn to MCF-7 and wt-ZIP7 + Zn to MCF-7 + Zn. * = *p* < 0.05, ** = *p* < 0.01.

**Fig. 4:**
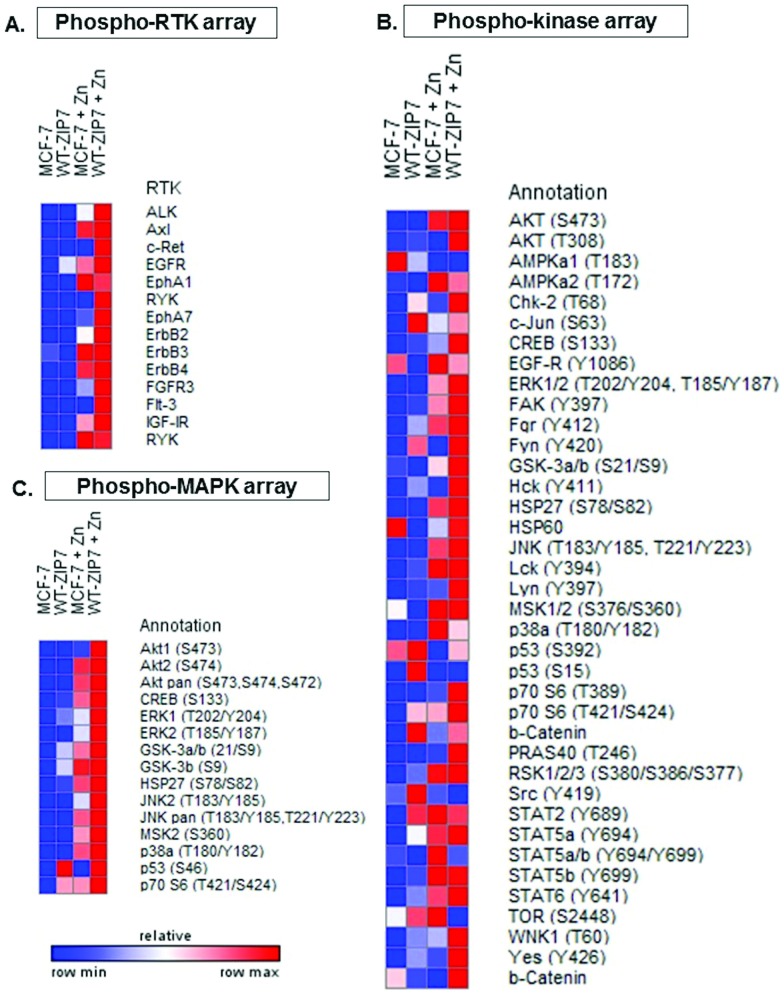
Cellular kinases activated by ZIP7 overexpression or zinc treatment. MCF-7 cells were transfected with or without wild-type ZIP7 and treated with or without zinc for 10 minutes. Tyrosine phosphorylation of selected RTKs and site-specific phosphorylation of selected kinases were determined using the phospho-RTK (A), phospho-kinase (B), and phospho-MAPK (C) arrays (R&D systems). Average densitometric values are presented as a spectrum of colour where blue and red colours represent the lowest and the highest values in the row according to the indicated scale.

We have previously reported the ability of CK2-mediated ZIP7 activation of zinc release from cellular stores to activate phosphorylation of tyrosine kinases.^5,10^ To further identify additional kinases that are phosphorylated as a result of ZIP7-mediated zinc release from cellular stores, we employed 3 different antibody arrays: the phospho-RTK, phospho-kinase, and phospho-MAPK arrays, which are able to indicate phosphorylation of up to 49 RTKs, 43 kinases, and 26 MAPKs, respectively. We tested these arrays with MCF-7 cells with or without transfection with wild-type ZIP7 and with or without zinc treatment for 10 minutes. Given that endogenous ZIP7 activates AKT within 10 minutes of zinc treatment (Fig. 1D), we performed zinc treatment for 10 minutes to allow sufficient time for ZIP7-mediated zinc release and downstream activation of zinc-stimulated effectors, with limited activation of the additional downstream pathways. To highlight the key kinases that were activated, only those with changes in density more than 10 000 units are indicated on the arrays with bar graphs also shown (Fig. 3A–C). Changes in the signal intensities of all the activated kinases are also presented as heat maps (Fig. 4A–C) and bar graphs (Fig. S2, ESI†).

Using the phospho-RTK arrays, only tyrosine phosphorylation of ErbB3 was detected in the control MCF-7 cells (Fig. 3A). ZIP7 overexpression resulted in an increase in EGFR tyrosine phosphorylation and a decrease in ErbB3 tyrosine phosphorylation (Fig. 3A and 4A). The arrays confirmed zinc treatment-induced tyrosine phosphorylation of EGFR, ErbB2, ErbB3, and ErbB4, confirming previous data,^10^ and further discovered the zinc-induced activation of ALK, EphA1, and RYK in comparison to control MCF-7 cells (Fig. 3A and 4A). In zinc-treated cells, wild-type ZIP7 transfection further enhanced tyrosine phosphorylation of ALK, EGFR, ErbB2, EphA7, and FGFR3, confirming that the activation of these kinases were influenced by ZIP7 (Fig. 3A and 4A).

The phospho-kinase arrays, enabled identification of the phosphorylation of individual kinase residues. Multiple kinases were already phosphorylated in control MCF-7 cells (Fig. 3B). Both ZIP7 overexpression alone and zinc treatment alone were able to induce phosphorylation of CREB (S133), p70 S6 (T421/S424), STAT2 (Y689), STAT5a (Y694), and WNK1 (T60) (Fig. 3B and 4B). In addition, c-Jun (S63) was phosphorylated only by ZIP7 overexpression, whereas AKT (S473), GSK-3α/β (S21/S9), HSP27 (S78/S82), JNK (T183/Y185, T221/Y223), p38α MAPK (T180/Y182), STAT5a/b (Y694/Y699), and STAT5b (Y699) were phosphorylated only by zinc treatment alone (Fig. 3B and 4B). Cells both transfected with wild-type ZIP7 and treated with zinc had a further increase in phosphorylation of CREB (S133), GSK-3α/β (S21/S9), HSP27 (S78/S82), p70 S6 (T421/S424), PRAS40 (T246), and WNK1 (T60) when compared to non-transfected zinc-treated cells (Fig. 3B and 4B), confirming ZIP7 mediation of the phosphorylation of these kinases. Due to the high variation of the data between the duplicates, only a small proportion of these increases were statistically significant (Fig. 3B). Noteworthy, one of the two replicates revealed an increase in AMPKα1 (T183) phosphorylation by 40 014 density units after zinc treatment (Fig. 3B). However, the other replicate instead showed a decrease by 14 351 density units. This kinase was therefore not included in further studies.

To confirm the phospho-kinase array data and investigate more MAPK isoforms, the phospho-MAPK arrays were also utilised. Without transfection or treatment, GSK-3α/β (S21/S9), HSP27 (S78/S82), and p70 S6 (T421/S424) were already phosphorylated (Fig. 3C). As a result of ZIP7 overexpression, GSK-3α/β (S21/S9) and p70 S6 (T421/S424) were further phosphorylated (Fig. 3C and 4C). Consistent with the phospho-kinase array results, zinc treatment alone induced phosphorylation of AKT1 (S473), CREB (S133), GSK-3α/β (S21/S9), HSP27 (S78/S82), JNK (T183/Y185, T221/Y223) and p70 S6 (T421/S424), with additional detection of AKT2 (S474) and ERK1 (T202/Y204) (Fig. 3C and 4C). ZIP7 transfection plus zinc treatment revealed further activation of CREB (S133), GSK-3α/β (S21/S9), HSP27 (S78/S82), and p70 S6 (T421/S424) (Fig. 3C and 4C).

**Fig. 5:**
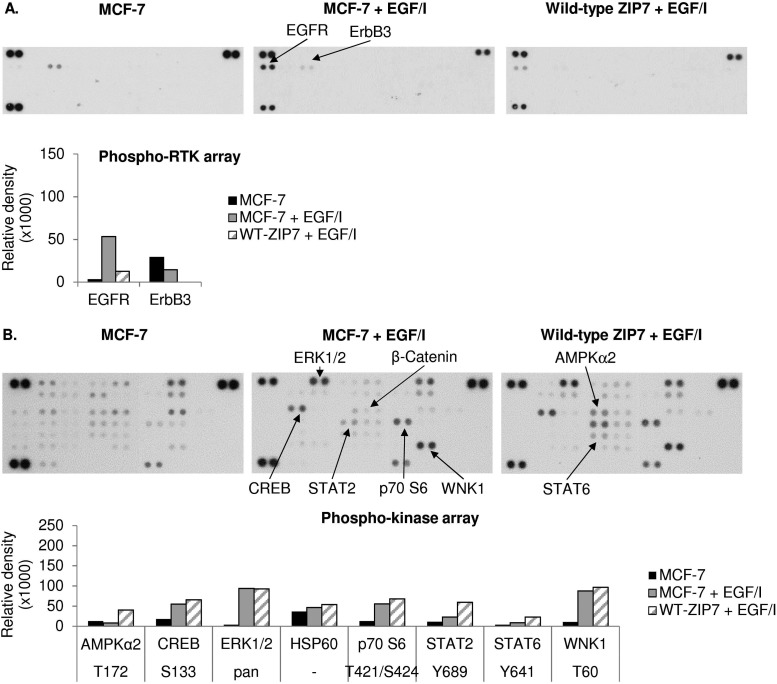
Activation of cellular kinases by ZIP7 overexpression or EGF and ionomycin treatment. MCF-7 cells were transfected with or without wild-type ZIP7 and treated with or without EGF plus ionomycin (EGF/I) for 10 minutes. Tyrosine phosphorylation of selected RTKs and site-specific phosphorylation of selected kinases were determined using the phospho-RTK (A) and phospho-kinase (B) antibody arrays (R&D systems). Signals for each kinase are presented as a pair of duplicate spots, with three pairs of dark reference spots on the upper left, upper right, and lower left corners for alignment. The kinases that show any changes in phosphorylation (>10 000 density units) in the non-transfected EGF/I-treated cells (MCF-7 + EGF/I) when compared to the non-transfected and non-treated cells (MCF-7), or in the transfected EGF/I-treated (wt-ZIP7 + EGF/I) when compared to the non-transfected EGF/I-treated cells (MCF-7 + EGF/I) are indicated. Average densitometric values for these kinases are shown in bar graphs. The experiments were performed once.

All the antibody array data together have revealed multiple kinases phosphorylated as a direct result of ZIP7-mediated zinc release from cellular stores, which are listed in Table 2. Interestingly, there is good agreement between the kinases activated by ZIP7 and those activated by zinc treatment. As the observed activation of p38α MAPK by the zinc treatment may have been indicative of an unphysiological zinc stimulation, we stimulated further phospho-RTK and phospho-kinase arrays with EGF and Ionomycin, which have previously been demonstrated to activate ZIP7-mediated zinc release.^5^ This stimulation did not show any RTK phosphorylation on the phospho-RTK arrays except EGFR, which might result from the direct activation by EGF independently of ZIP7-mediated zinc release (Fig. 5A). The phospho-kinase arrays confirmed the ZIP7-dependent phosphorylation of CREB, ERK1/2, p70 S6, STAT2, and WNK1, with additional increase in the total HSP60 level observed as a result of the stimulation (Fig. 5B). Importantly, p38α MAPK activation was not seen as a result of this treatment, suggesting possible cell stress induced by supra-physiologic zinc stimulation. Noteworthy, ZIP7 transfection in addition to stimulation with EGF plus Ionomycin further enhanced phosphorylation AMPKα2 (T172), CREB (S133), p70 S6 (T421/S424), STAT2 (Y689), and STAT6 (Y641) (Fig. 5B), confirming these kinases as downstream effectors of ZIP7.

**Table 2:** Downstream effectors of ZIP7-mediated zinc release and zinc treatment shows the kinases phosphorylated by either wild-type ZIP7 transfection or zinc treatment from the antibody arrays. Kinases with more than one isoform or modification are merged together. Kinases shown in bold are phosphorylated by both ZIP7 transfection and a treatment, either zinc plus sodium pyrithione or EGF plus ionomycin (EGF/I). Only kinases with changes in levels by more than 10 000 density units are listed

ZIP7 transfection		Zinc treatment		EGF/I treatment	
Receptor-tyrosine kinases (RTK) arrays					
**ALK**	EphA7	**ALK**	ErbB4	**EGFR**	
**EGFR**	FGFR3	**EGFR**	EphA1		
**ErbB2**		**ErbB2**	RYK		
		ErbB3			

Phospho-kinase and mitogen-activated protein kinases (MAPK) arrays					
**AKT**	p53	**AKT**	**JNK**	**CREB**	**p70 S6**
**AMPK**	**p70 S6**	**AMPK**	p38α	**ERK1/2**	**STAT2**
c-Jun	PRAS40	**CREB**	MAPK	HSP60	**WNK1**
**CREB**	**STAT2**	**GSK-3**	**p70 S6**		
**ERK1/2**	**STAT5**	**α/β**	**STAT5**		
**GSK-3α/β**	STAT6	**HSP27**	**WNK1**		
**HSP27**	**WNK1**				
**JNK**					

### GSK-3β and p70 S6 are key downstream effectors of ZIP7-mediated zinc release

**Fig. 6:**
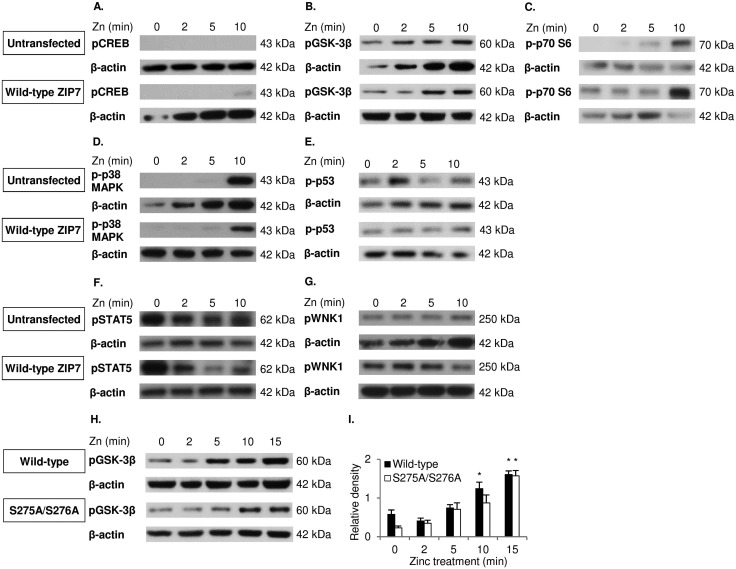
Investigation of ZIP7-dependent phosphorylation of GSK-3β, CREB, p38 MAPK, p53, p70 S6, STAT5 and WNK1. Immunoblotting using pS133 CREB (A), pS9 GSK-3β (B), pT421/S424 p70 S6 (C), pY180/Y182 p38 MAPK (D), pS392 p53 (E), pS726 STAT5 (F), and pT60 WNK1 (G) antibodies was performed in MCF-7 cells transfected with and without wild-type ZIP7 and treated with zinc for 0–10 minutes. Immunoblotting using the pS9 GSK-3β antibody was also performed on MCF-7 cells transfected with wild-type ZIP7 and the ZIP7 S275A/S276A mutant and treated with zinc for 0–15 minutes (H and I). Statistical significance is compared to time 0. * = *p* < 0.05.

To confirm the antibody array data, cells with and without ZIP7 transfection and/or 10 minutes of zinc treatment were probed with various antibodies by Western blotting. Phosphorylation of CREB (S133) (Fig. 6A), GSK-3β (S9) (Fig. 6B) and pT421/S424 p70 S6 (Fig. 6C) was apparently increased as a result of zinc treatment in the transfected cells when compared to the untreated cells, with the phospho-kinase levels being higher in the transfected cells than the untransfected cells when treated with zinc. In contrast, phosphorylation of p38 MAPK on Y180/Y182 was induced only by zinc, with no difference detected due to the ZIP7 transfection (Fig. 6D). Directly contradicting the phospho-array data, pS392 p53 (Fig. 6E), pS726 STAT5 (Fig. 6F), and pT60 WNK1 (Fig. 6G) did not show an apparent increase as a result of either ZIP7 overexpression or zinc treatment. Collectively, these results have confirmed CREB, GSK-3β and p70 S6 as downstream effectors of ZIP7-mediated zinc release from cellular stores.

To decipher the relationship of phosphorylation of GSK-3β on residue S9 with ZIP7 activation by phosphorylation on residues S275/S276, a course of zinc treatment was performed in cells transfected with either wild-type ZIP7 or the ZIP7 S276A/S276A mutant. GSK-3β was phosphorylated on residue S9 after 10 minutes of zinc treatment in cells transfected with wild-type ZIP7 compared to the delay of 15 minutes with the ZIP7 S275A/S276A mutant (Fig. 6H and I), confirming that this GSK-3β phosphorylation is dependent upon ZIP7 phosphorylation on residues S275/S276.

## D Conclusions

**Fig. 7:**
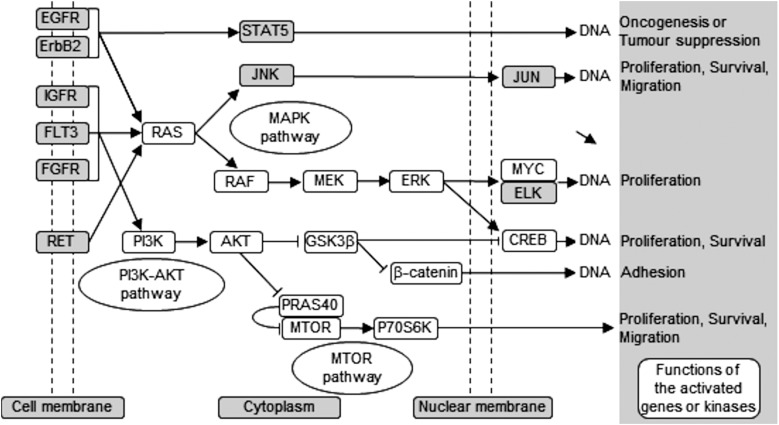
Schematic of ZIP7-mediated activation pathways. This schematic demonstrates carcinogenesis-related pathways that are activated downstream of ZIP7-mediated zinc release as observed from the phospho-array data (white box), showing activation of MAPK, PI3K-AKT, and mTOR pathways.

Using different phospho-kinase arrays, we have discovered multiple kinases phosphorylated as a direct result of ZIP7-mediated zinc release from intracellular stores (Fig. 3–5 and Table 2). Interestingly, many of these kinases are linked together in an integrated network of carcinogenesis-related pathways, involving MAPK, PI3K-AKT, and MTOR pathways (Fig. 7). In response to an extracellular stimulus, ZIP7 is activated by phosphorylation on residues S275 and S276, resulting in zinc release from intracellular stores,^5^ inhibition of protein tyrosine phosphatases,^6,24,25^ and thereby activation of RTKs as well as other cellular kinases,^10^ which may be triggered directly by zinc or indirectly by the zinc-activated RTKs. Here we confirm that activation of AKT is a major early response to ZIP7 activation,^5^ showing a significant increase within 5 minutes of a treatment (Fig. 1B) that stimulated phosphorylation of ZIP7 within 2 minutes (Fig. 1D). Interestingly, the observed inhibition of GSK-3β was not significant until 10 minutes after stimulation (Fig. 6H and I), suggesting that it may have been reliant on preceding AKT activation.^26^ However, zinc has been shown to be able to inhibit GSK-3β directly by triggering phosphorylation of it on residue S9.^27^

The activation of AKT is known to result from the inhibitory effect of zinc on protein tyrosine phosphatases^24^ or indirectly from zinc-induced phosphorylation of RTKs such as IGF-1R.^27^ Importantly, silencing protein tyrosine phosphatase 1B, a notable zinc-modulated phosphatase^25^ which is involved in EGFR, IGF-1R and Src signalling,^10^ also enhances AKT phosphorylation.^28^ We therefore propose that the ZIP7-mediated release of zinc from intracellular stores is sufficient to activate multiple signalling pathways involved in encouraging growth and proliferation, especially those known to be aberrantly activated in diseases such as cancer.^29–31^

Using the phospho-arrays, we also discovered that CREB and p70 S6 kinases were both consistently activated by ZIP7-mediated zinc release (Fig. 3 and 4) which was amplified further by zinc (Fig. 4). Both CREB and p70 S6 kinase are known to be phosphorylated in response to zinc using the ERK1/2^32^ and the EGF-dependent PI3K pathways,^33^ respectively. In neuroblastoma cells, zinc-induced phosphorylation of p70 S6 kinase is associated with the pathogenesis of Alzheimer's disease,^34^ and in pancreatic cancer cells, ZIP4 overexpression results in CREB phosphorylation, which in turn induces cyclin D1 expression and thereby cell proliferation.^35^ Importantly, phosphorylation of CREB,^36^ GSK-3β,^37^ and p70 S6^38^ have all been shown to contribute to cell proliferation and survival, linking ZIP7 to these major signalling pathways.

ZIP7, a gatekeeper for zinc release from cellular stores and a hub for phosphorylation of tyrosine kinases,^18^ actively participates in carcinogenesis,^15^ including but not limited to the development of endocrine resistance in breast cancer.^10^ Furthermore, using Kaplan–Meier analysis, we have previously reported that high ZIP7 gene expression is associated with decreased relapse-free survival in breast cancer patients when compared to low ZIP7 expression cases.^39^ These findings therefore will help us to understand the consequences of action of ZIP7 in order to develop a novel strategy for targeting ZIP7 in cancer patients.

We have developed a monoclonal antibody and demonstrated that it specifically recognises the active form of ZIP7. Interestingly, this antibody produced an 8-kDa mobility shift compared to total ZIP7 (Fig. 1A), consistent with the presence of additional phosphorylated residues in ZIP7.^40^ Furthermore, our investigation into the phosphorylation of residues S293 and T294 in ZIP7 is consistent with the presence of hierarchical phosphorylation,^41^ a common feature of CK2 phosphorylation patterns.^42^ We determined that all four residues, S275, S276, S293, and T294 in ZIP7 were required for full activation of ZIP7-mediated zinc release (Fig. 2), suggesting a complexity yet to be deciphered. The development of the pZIP7 antibody now provides a useful tool for assessing the activation state of zinc signalling in cells, particularly a measure of the activation of the multiple signalling pathways downstream of ZIP7-mediated zinc release, such as AKT, PI3K, MAPK and MTOR. Additionally, as there is currently no reliable biomarker for total body zinc status assessment,^43^ and ZIP7 is ubiquitously expressed, especially in immune cells, this pZIP7 antibody should be investigated for efficacy in this area as a reliable biomarker of zinc status to benefit those with zinc deficiency.
