# Supplementary material for: Phosphorylation of zinc channel ZIP7 drives MAPK, PI3K and mTOR growth and proliferation signalling
Source: Metallomics. 2017 Feb 8;9(5):471–81. doi: 10.1039/c6mt00286b (PMC5451890; doi:10.1039/c6mt00286b)
Supplement: Supplementary file 1 [file MT-009-C6SC04798J-s001.pdf]

Electronic Supplementary Material (ESI) for Metallomics.
This journal is © The Royal Society of Chemistry 2017

Supplementary Figure 1

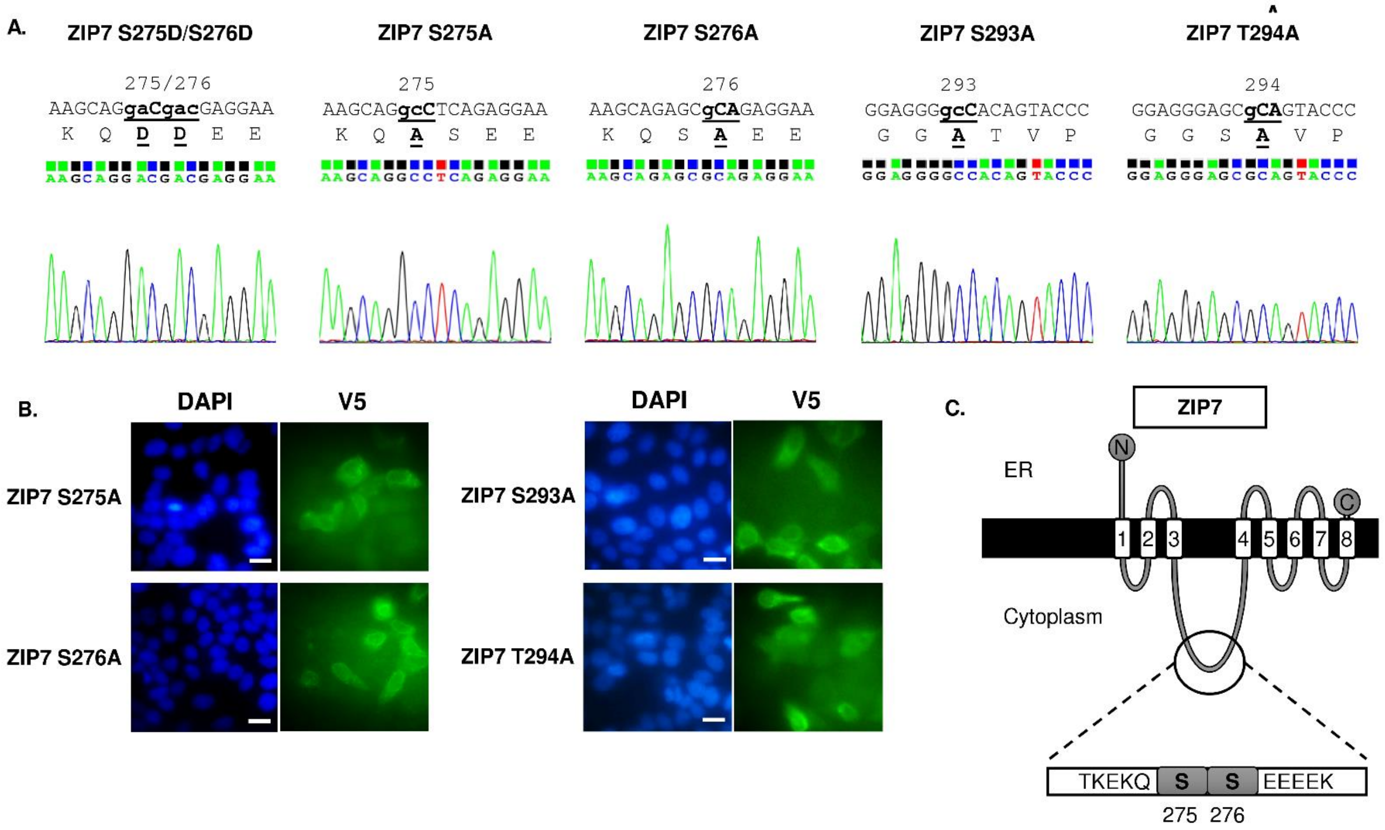

Supplementary figure 1

A) DNA sequencing confirms that a ZIP7 recombinant construct with a C−terminal V5 tag [16] is correctly mutated to create S275D/S276D, S275A, S276A, S293A, and T294A mutants.

B) Robust transfection of ZIP7 S275A, S276A, S293A, and T294A mutants is revealed by immunofluorescence using a V5 antibody conjugated to Alexa Fluor 488. Scale bar, 12 µm.

C) A pS275/S276 ZIP7 antibody was created with epitope TKEKQ pS pS EEEEK, which is located in the cytoplasmic loop between TM3 and TM4 of ZIP7.

Supplementary Figure 2

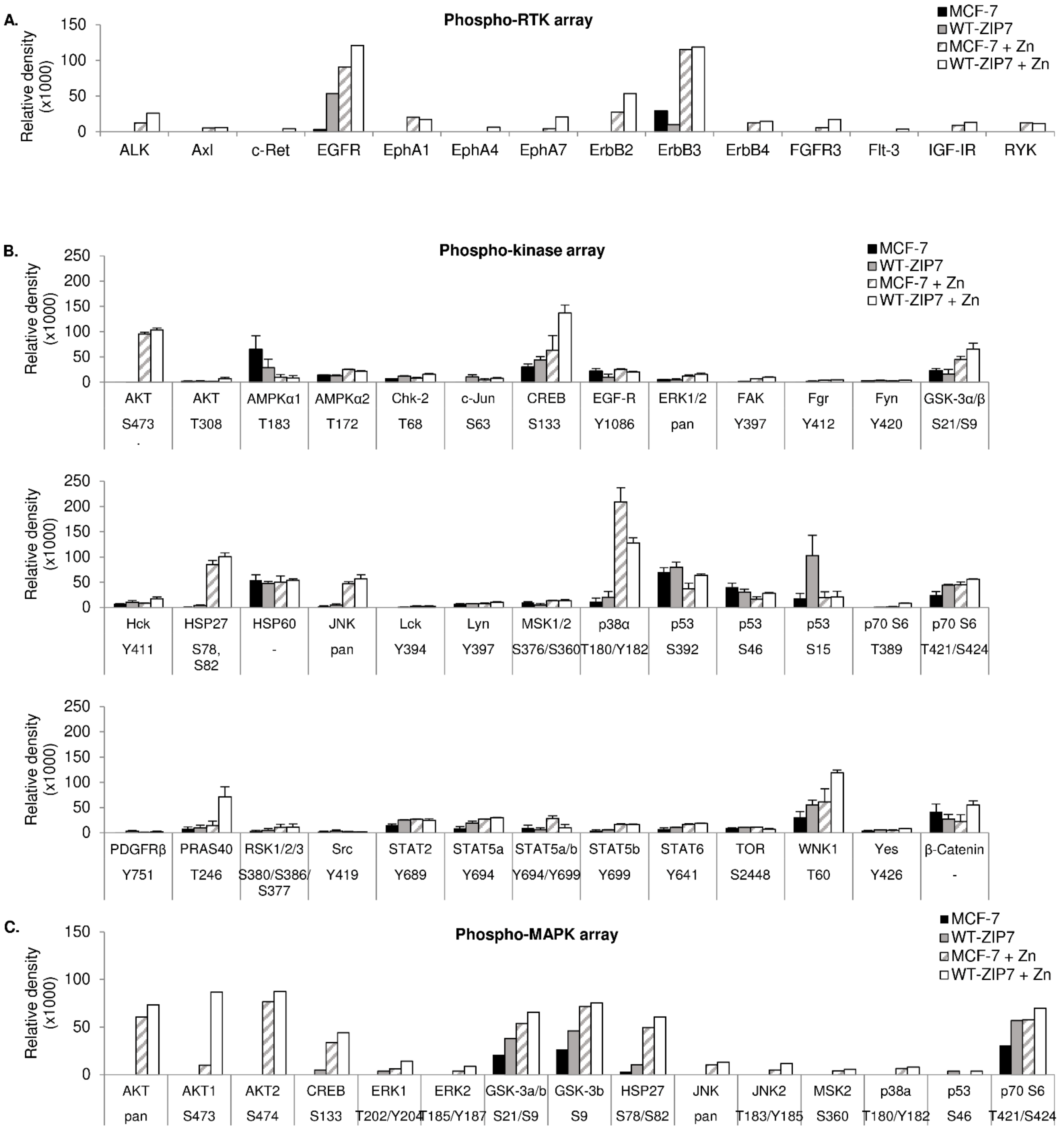

MCF−7 cells were transfected with wild−type ZIP7 and treated with zinc for 10 minutes. Tyrosine phosphorylation of selected RTKs and site−specific phosphorylation of selected kinases were determined using the phospho−RTK (A), phospho−kinase (B), and phospho−MAPK (C) arrays (R&D Systems). Heat maps were generated using a GENE−E matrix visualization and analysis platform (The Board Institute). Densitometric values in relation to other samples for each kinase are presented as a spectrum of colour where blue colour represents the lowest value in the row and red colour represents the highest value in the row according to the indicated scale, irrespective of absolute signal intensities.
